# Esophageal Varices: Pathophysiology, Approach, and Clinical Dilemmas

**DOI:** 10.1155/2012/795063

**Published:** 2012-11-05

**Authors:** Nir Hilzenrat, Averell H. Sherker

**Affiliations:** ^1^McGill University, Montreal, QC, Canada; ^2^Liver Diseases Research Branch, National Institute of Diabetes and Digestive and Kidney Diseases, Bethesda, MD, USA

Portal hypertension is one of the most significant complications of both acute and chronic liver diseases. It generally develops as a result of an increase in vascular resistance at the prehepatic, intrahepatic, or postherpetic level. An increase in portal blood flow may also contribute. The dominant cause of portal hypertension relates to liver cirrhosis which increases resistance through the hepatic sinusoids. Gastroesophageal varices are the most important clinical manifestation of this syndrome and are associated with a high risk of upper gastrointestinal hemorrhage and its attendant high mortality.

 This special issue includes nine evidence-based reviews. They discuss the pathophysiology of portal hypertension as well as its clinical manifestations and management. Selected topics and controversies related to esophageal varices are covered, including noninvasive diagnostic methods, bleeding prophylaxis in adults and children, rescue treatments, and the clinical dilemma of portal vein thrombosis.

 H. Maruyama and O. Yokosuka review the current concepts of the pathophysiology of portal hypertension and esophageal varices. Portal hypertension is initially caused by distortion of the hepatic vascular bed, which in turn leads to increased resistance to portal blood flow. This phenomenon is associated with intrahepatic endothelial dysfunction with a resultant imbalance between vasodilators such as nitric oxide and prostaglandins vasoconstrictors including endothelin. An important consequence of increased resistance to portal blood flow is splanchnic vasodilatation with consequent sodium and water retention. As a result of the plasma's expansion, and the reduction in peripheral resistance, a hyperdynamic circulation develops. Consequently, there is a significant increase in the blood flow through the portal vein which further contributes to portal hypertension. Esophageal varices appear and may bleed when the HVPG exceeds 12 mmHg. 

A comprehensive review of the clinical manifestations of portal hypertension is presented by S. A. Al-Busafi et al. Portal hypertension is a common clinical syndrome defined as the elevation of hepatic venous pressure gradient (HVPG) above 5 mmHg. Its gastrointestinal manifestations include the development of esophageal varices, gastric varices, and intestinal vasculopathy. Approximately 5–15% of cirrhotics develop esophageal varices annually. The majority of patients with cirrhosis are expected to develop this condition over their lifetime. Beyond its gastrointestinal effects, portal hypertension may also affect other vital organs resulting in extrahepatic manifestations. 

Y.-I. Chen and P. Ghali present an overview of strategies to prevent and manage portal hypertension. The one-year rate of first variceal hemorrhage is 5% for small varices and 15% for large varices. Six-week mortality rate following an episode of bleeding varies between 15 and 20%. Clearly, a strategy of prophylaxis to prevent the first episode of bleeding may reduce morbidity and mortality. In this respect, nonselective beta blockers and new types of beta blockers play a major role. The overall approach and the current pharmacological therapy of acute hemorrhage and of recurrent bleeding (i.e., secondary prophylaxis) are based on understanding the pathophysiology of esophageal varices.

Early diagnosis of esophageal varices prior to the first episode of bleeding is essential. Studies of primary prophylaxis clearly show that the risk of first variceal haemorrhage can be reduced significantly. Upper GI endoscopy remains the gold standard for screening, but this test is not without its own limitations. K. Rye et al. review the utility of noninvasive tests to predict esophageal varices. Unfortunately, current clinical, biochemical, and radiological parameters are not accurate enough to detect varices without a screening endoscopy, but assessment of systemic hemodynamics and other serum markers may hold promise for the future. 

 Variceal rupture is governed by Laplace's law. Increased wall tension is the end result of increased intravariceal pressure, increased diameter of the varices, and reduced wall thickness. The variceal wall thickness can be evaluated visually by the presence of red wale markings. These markings reflect areas where the wall is especially thin. Variceal rupture often occurs at the level of the gastroesophageal junction where the varices are very superficial and thus have thinner walls. S. A. Al-Busafi et al. discuss the role of endoscopic management of esophageal varices. They emphasized the fact that gastroscopy allows direct visualization and is an excellent tool to assess the size and the presence of high risk stigmata of bleeding. A debate exists as to whether a pharmacologic or an endoscopic approach is the best method of primary prophylaxis. It was shown that both modalities are effective in minimizing the risk of a first episode of bleeding in patients with cirrhosis and large esophageal varices, independently of the presence of red signs. However, the endoscopic approach is the treatment of choice whenever the patient is unable to tolerate beta blockers. Acute variceal bleeding in patients with cirrhosis indicates decompensation and a high risk of death. Initial treatment for these patients includes volume resuscitation and administration of vasoactive drugs and antibiotics. Emergency endoscopic variceal ligation, one of the cornerstones of management, should be performed within the first 12 hours of hospital admission. 

G. Pomier-Layrargues et al. highlight the role of transjugular intrahepatic portosystemic shunt (TIPS) in the treatment of acute esophageal varices bleeding. The clinical trials indicate that the TIPS procedure is not a first line therapy for variceal bleeding but can be used when medical and endoscopic treatments fail, either in the acute situation or to prevent variceal rebleeding. However, careful selection of patients is mandatory before the TIPS procedure. Clinical followup is essential to detect and treat complications that may result from TIPS stenosis, which can be minimized by using covered stents. Followup is also required to monitor for worsening portosystemic encephalopathy. In severe cases of encephalopathy, reduction or occlusion of the shunt may be warranted.

 The current first line pharmacologic and endoscopic therapies fail to control bleeding in approximately 10–15% of patients. Rescue therapies, which include balloon tamponade or TIPS, have many limitations and are contraindicated in some cases. A novel, emerging therapy is reviewed by F. Maufa and F. H. Al-Kawas. Placement of a fully covered self-expandable metallic stent can be used to control bleeding in cases of refractory esophageal hemorrhage. The removable stent can be left in place for as long as two weeks, allowing for improvement in liver function while a more definitive treatment can be planned semielectively. 

 Portal hypertension in children represents a particular challenge in both diagnosis and management. A. Costaguta and F. Alvarez describe the progress that has been achieved recently in the treatment of children with portal hypertension. Two main factors influence therapeutic decisions: the age of the patient and the etiology of the liver disease. In this special issue, one can find a summary of the current knowledge and an expert opinion on the subject.

Finally, nonneoplastic portal vein thrombosis (PVT) can be found in up to 25% of individuals with liver cirrhosis. The major risk factor of having PVT is severe liver disease and portal hypertension. Recently, it was found that procoagulant imbalance in individuals with advanced liver disease contributes to the development of PVT. The clinical impact of PVT on liver function is not clear. Nevertheless, it is a predictive factor for mortality among cirrhotics, independent of MELD score. PVT may be the cause of various life-threatening conditions. It increases portal hypertension and the risk of variceal bleeding. It may also extend into the superior mesenteric vein causing intestinal ischemia. The optimal management of PVT in individuals with cirrhosis is currently not addressed in any consensus publication or practice guidelines. G. Huard and M. Bilodeau explore the different aspects of PVT management including the potential risks and benefits of anticoagulation. 

This special issue provides an excellent overview of one of the more complicated topics in the field of liver disease. It covers current concepts of the clinical and pathophysiological aspects of portal hypertension, management of the condition, along with emerging diagnostic and therapeutic modalities, and clinical controversies. We would like to congratulate the authors for their superb scientific manuscripts.

*Nir Hilzenrat* *Nir Hilzenrat* *Averell H. Sherker* *Averell H. Sherker*

